# An Analysis of Biologic Therapies in Patients With Asthma and Chronic Rhinosinusitis

**DOI:** 10.7759/cureus.30017

**Published:** 2022-10-07

**Authors:** Mitchell R Gore, Ethan Fung, Michael Tao

**Affiliations:** 1 Otolaryngology - Head and Neck Surgery, State University of New York Upstate Medical University, Syracuse, USA; 2 Otolaryngology, State University of New York Upstate Medical University, Syracuse, USA

**Keywords:** improving pulmonary function, atopic asthma, allergic asthma, paranasal sinusitis, biologic treatment

## Abstract

Background

Asthma, Allergic rhinitis (AR), Chronic Obstructive Pulmonary Disease (COPD), Eczema, and Chronic Rhinosinusitis with Sinonasal Polyposis (CRSwNP) are illnesses often characterized by type 2 (T2) inflammation, wherein T helper (Th) cells release pro-inflammatory cytokines such as IL (interleukin)-4, IL-5, IL-9, and IL-13. This response may also promote the production of IgE and an increase in/activation of serum eosinophils. In the aforementioned type 2 inflammatory diseases, this immune response can cause excess mucous production, inflammation of the airways, other atopic responses when patients are exposed to certain environmental allergic triggers. Relatively new biologic monoclonal antibody therapies such as dupilumab (blocks IL-4 and IL-13), benralizumab (blocks IL-5), mepolizumab (blocks IL-5), and omalizumab (blocks IgE Fc/fragment of crystallization region) offer novel therapeutic targets that more specifically and directly block type 2 inflammatory responses.

Methods

To examine the effect of monoclonal antibody biologic therapies on patient indicators of type 2 inflammation, a retrospective analysis of 193 patients on biologic therapy was conducted, and these patients were compared to 48 control patients with type 2 inflammatory diseases who did not initiate biologic therapy. Total Lund-MacKay radiographic score, FEV1 (forced expiratory volume in the first second), FEF25-75 (forced expiratory flow from 25-75% of the forced vital capacity curve), annualized pulmonary exacerbations, oral corticosteroid dose, and serum eosinophils were recorded at baseline (zero months), and at three, six, nine, and twelve months after initiation of biologic therapy. Least squares mean data and the percent change from the baseline of least squares mean for the biologic and control groups were compared.

Results

Omalizumab was the most common biologic therapy prescribed. Control patients were younger than patients who initiated biologic therapy. Patients on biologic therapy had statistically significant reductions in Lund-MacKay score, improvements in FEV1 and FEF25-75, reductions in serum IgE levels, and reductions in serum Eosinophils. Patients on biologic therapy also had statistically significant reductions in annualized pulmonary exacerbations and oral corticosteroid dose compared to controls.

Conclusions

Patients with a variety of type 2 inflammatory conditions appear to have significant improvements in lung function, radiographic sinusitis, and serum markers of type 2 inflammation after initiation of biologic therapy versus controls. These therapeutic medications appear to significantly improve type 2 inflammatory disease course in patients who can tolerate these medications.

## Introduction

Omalizumab, an anti-immunoglobulin (Ig)E humanized antibody, was approved over a decade ago as the first biological drug for poorly controlled asthma. Omalizumab reduces the atopic response to antigens mediated by IgE which can contribute to exacerbations in allergic asthma [1]. Further studies have demonstrated the efficacy of omalizumab in other type 2 inflammatory diseases, with studies showing that chronic rhinosinusitis with nasal polyposis (CRSwNP) patients treated with omalizumab showed improved radiographic Lund-MacKay scores, improved nasal polyposis on endoscopy, reductions in symptom scores, and reduced intranasal steroid needs [2-9]. Omalizumab has also been shown to be effective in chronic urticaria and has been used in patients with allergic rhinitis, anaphylaxis, angioedema, non-atopic asthma, atopic dermatitis, and other inflammatory/allergic diseases. Since the approval of omalizumab, other biologic monoclonal antibody therapies such as mepolizumab and benralizumab (which block IL-5 signaling) and dupilumab (which blocks IL-4 and IL-13) have been introduced [6-8]. These biologics have also been shown to improve symptom scores and inflammatory markers in patients with eosinophilic asthma, allergic rhinitis, chronic rhinosinusitis with and without nasal polyps, and urticaria. Omalizumab has been shown to improve daily nasal symptom score and daily ocular symptom score and the need for medication use in patients with allergic rhinitis, and omalizumab has been shown to improve nasal congestion and nasal polyp scores, Sinonasal Outcome test (SNOT-22), sense of smell, and total nasal symptom scores (TNSS), and to improve UPSIT (University of Pennsylvania Smell Identification Test) scores in patients with CRSwNP [5, 10-12]. This retrospective study aimed to examine the effect of biologic therapy on serum markers of inflammation and sinus radiographic and pulmonary function test parameters in patients treated with monoclonal antibody therapy vs. controls.

## Materials and methods

Between January and August of 2022, the SUNY Upstate medical record was queried for all patients treated with omalizumab, benralizumab, mepolizumab, and dupilumab for any indication. A total of 250 patients were identified, and after eliminating patients with insufficient data, 193 patients treated with biologic therapy for at least 12 months were identified, along with 48 control patients who were prescribed one of the aforementioned biologic drugs but who were unable to initiate therapy due to adverse or allergic reaction to the medication or due to insurance denial. Given the retrospective data the control group was not age or gender-matched.

Patients were included if they were prescribed one of the aforementioned biologic drugs and had longitudinal follow-up for the outcome measures noted below. Patients were excluded if they had insufficient longitudinal data on the outcome measures listed below. The indication for monoclonal antibody treatment in all of the pediatric patients was asthma. The baseline time (month 0) was recorded as the date of the initiation of biologic therapy for the experimental group, and as the date of the initial prescription in the control group.

Demographic data on sex, age, and diagnosis were recorded for the treatment and control groups. The medical record was searched for data on Lund-MacKay score [13-15], FEV1 (forced expiratory volume in the first second), FEF25-75 (forced expiratory flow from 25-75% of the forced vital capacity curve), serum IgE, annualized pulmonary exacerbations (pulmonary exacerbations per previous twelve month period), oral daily steroid dose, SNOT-22 score, ACQ-5 (Asthma Control Questionnaire-5) score, ACT (Asthma Control Test) score, NOSE score (Nasal Obstruction Symptom Evaluation survey score), need for functional endoscopic sinus surgery (FESS), FENO (fractional excretion of nitric oxide), and serum eosinophils.

Patient data on the above variables was compiled at months zero, three, six, nine, and twelve after the initiation of biologic therapy for the treatment group and after the initial prescription date for the control group. The change in the raw data over time as well as the percent change from the baseline of the least squares mean of the data for each variable was plotted. Patient data was de-identified and retrospective and this study was approved by the SUNY-Upstate Institutional Review Board (1829130-1).

Statistical analyses

Patient data were compiled in Microsoft Excel (Microsoft Corporation, Redmond, Washington, USA) and the data were analyzed using XLSTAT (Addinsoft, Paris, France). Continuous variables were analyzed using the Unpaired Student’s t-test for comparison of means and one-way analysis of variance (ANOVA) for comparison between groups. The level of statistical significance was set at p < 0.05.

## Results

Table 1 shows the patient characteristics for each group. The average age of the treatment group was 48.79 +/- 20.40 years, while the average age of the control group was 38.96 +/- 23.5 years (p=0.0041). Body mass index (BMI) was 31.85 +/- 12.14 in the biologic treatment group and 29.63 +/- 7.12 in the control group (p=0.23). In the treatment group, there were 84 male and 109 female patients, while in the control group there were 23 male and 25 female patients (p=0.63). In the biologic treatment group, there were 147 white patients, 30 black patients, 11 Hispanic patients, four Asian patients, and one Native American patient, while in the control group there were 31 white patients, 14 black patients, and three Hispanic patients (p=0.23). The primary diagnosis in the experimental group was asthma in 147 patients, CRSwNP in 21, Chronic Obstructive Pulmonary Diseas (COPD) in eight, eczema in 12, and anaphylaxis in five. In the control group, the primary diagnosis was asthma in 41 patients, CRSwNP in two, eczema in four, and COPD in one (p=0.57). In the treatment group, omalizumab was the most common biologic, with 87 patients, while 68 were treated with dupilumab, 16 were treated with benralizumab, and 22 were treated with mepolizumab. Sufficient data for analysis was not available for SNOT-22, ACT, or NOSE scores, or the need for FESS or FENO. Sufficient data was not available for Lund-MacKay score at six and nine months, or for IgE at nine or twelve months.

**Table 1:** Patient characteristics for the study patients. BMI: Body mass index; COPD: Chronic obstructive pulmonary disease; CRSwNP: Chronic rhinosinusitis with nasal polyposis. Age and BMI reported as Mean ± Standard Deviation.

	Biologic Group	Control Group	
Age	48.79 +/- 20.40	38.96 +/- 23.5	p=0.0041
Male	84 (43.52%)	23 (47.92%)	p=0.63
Female	109 (56.48%)	25 (52.08%)	
BMI	31.85 +/- 12.14	29.63 +/- 7.12	p=0.23
White	147 (76.17%)	31 (64.58%)	p=0.23
Black	30 (15.54%)	14 (29.17%)	
Hispanic	11 (5.70%)	3 (6.25%)	
Asian	4 (2.07%)		
Native American	1 (0.52%)		
asthma	147 (76.17%)	41 (85.42%)	p=0.57
CRSwNP	21 (10.88%)	2 (4.17%)	
eczema	12 (6.21%)	4 (8.33%)	
COPD	8 (4.15%)	1 (2.08%)	
anaphylaxis	5 (2.59%)		
omalizumab	87 (45.07%)		
dupilumab	68 (35.23%)		
benralizumab	16 (8.29%)		
mepolizumab	22 (11.40%)		

Table 2 shows the least squares mean values (+/- standard deviation) for the biologic and control groups and p-values at zero, three, six, nine, and twelve months for Lund-MacKay score, FEV1, FEF25-75, serum IgE, annualized pulmonary exacerbations, daily steroid dose, and serum eosinophils.

**Table 2:** Least squares mean values (+/- standard deviation) and p-values at zero, three, six, nine, and twelve months for Lund-MacKay score, FEV1, FEF25-75, IgE, annulaized pulmonary exacerbations, daily steroid dose, and eosinophils for biologic and control groups. FEF25-75: forced expiratory flow from 25% to 75% of forced vital capacity curve; FEV1: forced expiratory volume in the first second; IgE: immunoglobulin E; IU: international units; LM: Lund-MacKay; SD: standard deviation.

Outcome	Month	Biologic group(SD)	Control group(SD)	p-value
Lund-Mackay score	0	8.45(6.61)	5.42(5.08)	p=0.023
	3	1.17(0.27)	14(13)	p<0.0001
	12	5.26(4.24)	7.21(7.29)	p=0.087
FEV1(Liters)	0	2.61(0.79)	2.28(1.13)	p<0.0001
	3	2.76(0.84)	1.98(0.98)	p<0.0001
	6	2.77(0.83)	2.07(1.02)	p<0.0001
	9	2.77(0.83)	2.03(1.03)	p<0.0001
	12	2.42(0.72)	2.15(1.05)	p=0.014
FEF25-75(Liters/second)	0	2.31(1.19)	2.19(1.21)	p=0.61
	3	2.49(1.31)	1.77(1.03)	p=0.0004
	6	2.74(1.46)	1.96(1.16)	p=0.004
	9	2.53(1.27)	1.9(1.2)	p=0.0081
	12	2.15(1.35)	1.95(1.15)	p=0.32
IgE(IU/milliliter)	0	1001(1299)	608.2(858.2)	p=0.22
	3	670.63(839.37)	972(1428)	p=0.11
	6	423.5(573.5)	854.83(1295.17)	p=0.0018
Annualized asthma exacerbations	0	1.53(2.97)	2.42(1.38)	p<0.0001
	3	0.45(0.75)	2.32(1.18)	p<0.0001
	6	0.31(0.59)	2.45(1.35)	p<0.0001
	9	0.29(0.51)	2.63(1.33)	p<0.0001
	12	0.37(0.73)	2.5(1.3)	p<0.0001
Daily oral corticosteroid dose (milligrams)	0	22.41(45.59)	28.76(13.24)	p=0.03
	3	6.03(12.03)	28.29(13.71)	p<0.0001
	6	4.98(9.98)	28.92(13.08)	p<0.0001
	9	5.35(10.65)	27.25(13.75)	p<0.0001
	12	5.26(9.74)	25.76(13.24)	p<0.0001
Serum eosinophils (x1000/microliter)	0	0.33(0.33)	0.25(0.51)	p=0.16
	3	0.26(0.29)	0.24(0.51)	p=0.69
	6	0.28(0.32)	0.38(0.77)	p=0.24
	9	0.27(0.31)	0.39(0.79)	p=0.094
	12	0.28(0.32)	0.4(0.8)	p=0.10

Table 3 shows the percent change from baseline/100 of the least squares mean values (+/- standard deviation) for the biologic and control groups and p-values at three, six, nine, and twelve months for Lund-MacKay score, FEV1, FEF25-75, serum IgE, annualized pulmonary exacerbations, daily steroid dose, and serum eosinophils.

**Table 3:** Percent change from baseline/100 of the least squares mean value (+/- standard deviation) and p-values at three, six, nine, and twelve months for Lund-MacKay score, FEV1, FEF25-75, IgE, annualized pulmonary exacerbations, daily steroid dose, and eosinophils for the biologic and control groups. FEF25-75: forced expiratory flow from 25% to 75% of forced vital capacity curve; FEV1: forced expiratory volume after one second; IgE: immunoglobulin E; LS: least squares; SD: standard deviation.

Outcome	Month	Biologic group (SD)	Control group(SD)	p-value
Lund-Mackay LS mean percent change from baseline/100	0	0	0	
	3	-0.86(0.66)	1.58(0.88)	p<0.0001
	12	-0.38(0.22)	0.33(0.17)	p<0.0001
FEV1 LS mean percent change from baseline/100	0	0	0	
	3	0.06(0.12)	-0.13(0.05)	p<0.0001
	6	0.06(0.12)	-0.09(0.03)	p=0.0014
	9	0.06(0.12)	-0.09(0.03)	p<0.0001
	12	-0.08(0.16)	-0.06(0.02)	p<0.0001
FEF25-75 LS mean percent change from baseline/100	0	0	0	
	3	0.08(0.1)	-0.19(0.16)	p<0.0001
	6	0.19(0.2)	-0.11(0.1)	p<0.0001
	9	0.1(0.1)	-0.13(0.11)	p<0.0001
	12	-0.07(0.07)	-0.11(0.02)	p<0.0001
IgE LS mean percent change from baseline/100	0	0	0	
	3	-0.33(0.53)	0.6(1.2)	p<0.0001
	6	-0.58(0.98)	0.41(0.84)	p<0.0001
Annualized asthma exacerbations LS mean percent change from baseline/100	0	0	0	
	3	-0.71(0.21)	-0.04(0.04)	p<0.0001
	6	-0.8(0.22)	0.01(0.001)	p<0.0001
	9	-0.81(0.24)	0.09(0.1)	p<0.0001
	12	-0.76(0.2)	0.03(0.02)	p<0.0001
Daily oral corticosteroid dose LS mean percent change from baseline/100	0	0	0	
	3	-0.73(0.23)	-0.02(0.01)	p<0.0001
	6	-0.78(0.23)	0.01(0.005)	p<0.0001
	9	-0.76(0.23)	-0.05(0.12)	p<0.0001
	12	-0.76(0.23)	-0.1(0.2)	p<0.0001
Serum eosinophils LS mean percent change from baseline/100	0	0	0	
	3	-0.22(0.08)	0.04(0.11)	p<0.0001
	6	-0.15(0.08)	0.52(1.08)	p<0.0001
	9	-0.18(0.1)	0.58(1.12)	p<0.0001
	12	-0.16(0.07)	0.61(1.19)	p<0.0001

**Figure 1:**
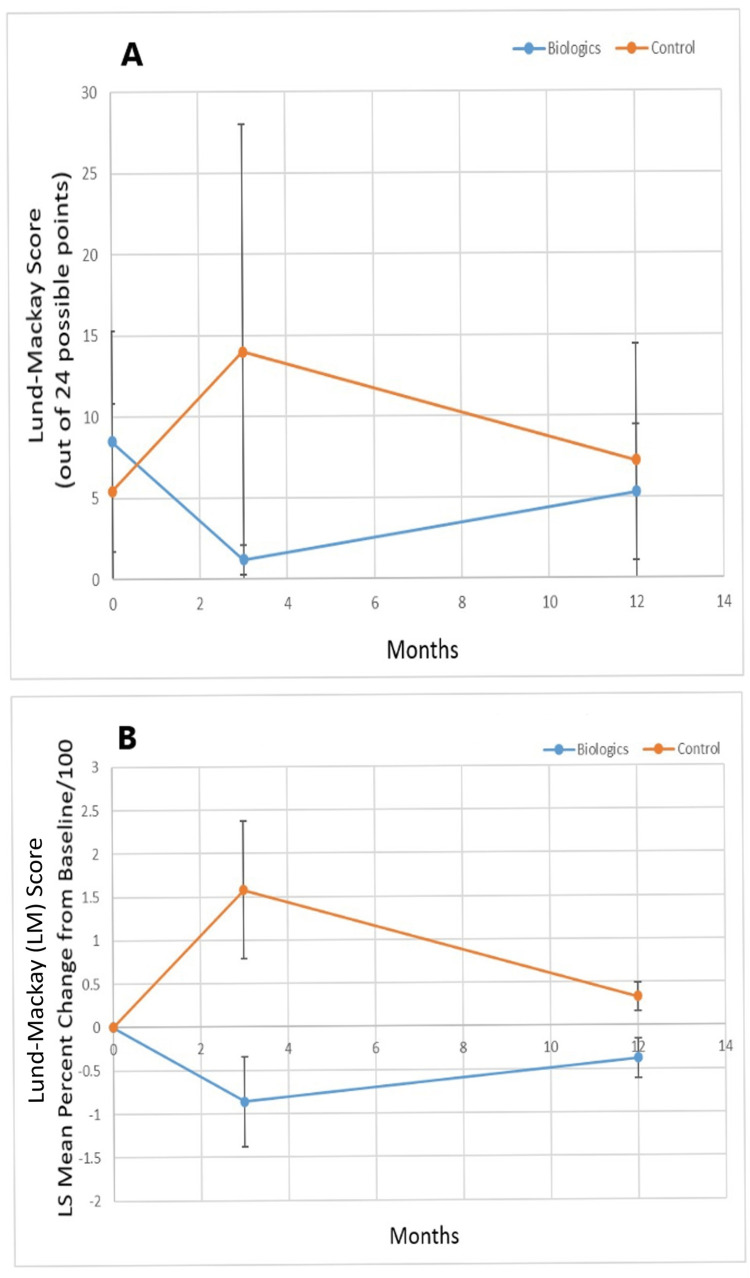
A. Least squares mean Lund-Mackay score (out of 24 maximum points, increased score indicates more severe radiographic sinus disease) over the study period for biologic versus control group and B. percent change of the least squares mean of Lund-Mackay score from baseline over the study period for biologic versus control group. LM: Lund-Mackay; LS: Least squares.

Figure 1A shows the Lund-MacKay score (out of 24 points) for the biologic versus control groups, while Figure 1B shows the percent change from baseline of the least squares mean value for the Lund-MacKay score for the biologic and control groups. The baseline Lund-MacKay score was higher for the biologic treatment group (p=0.023) but was lower for the biologic treatment group at three months (p<0.0001) and twelve months (p=0.087) vs. the control group. There was a statistically significant improvement (reduction) in total Lund-MacKay score least squares mean percent change from baseline for the biologic group vs. the control group at three and twelve months (p<0.0001).

**Figure 2:**
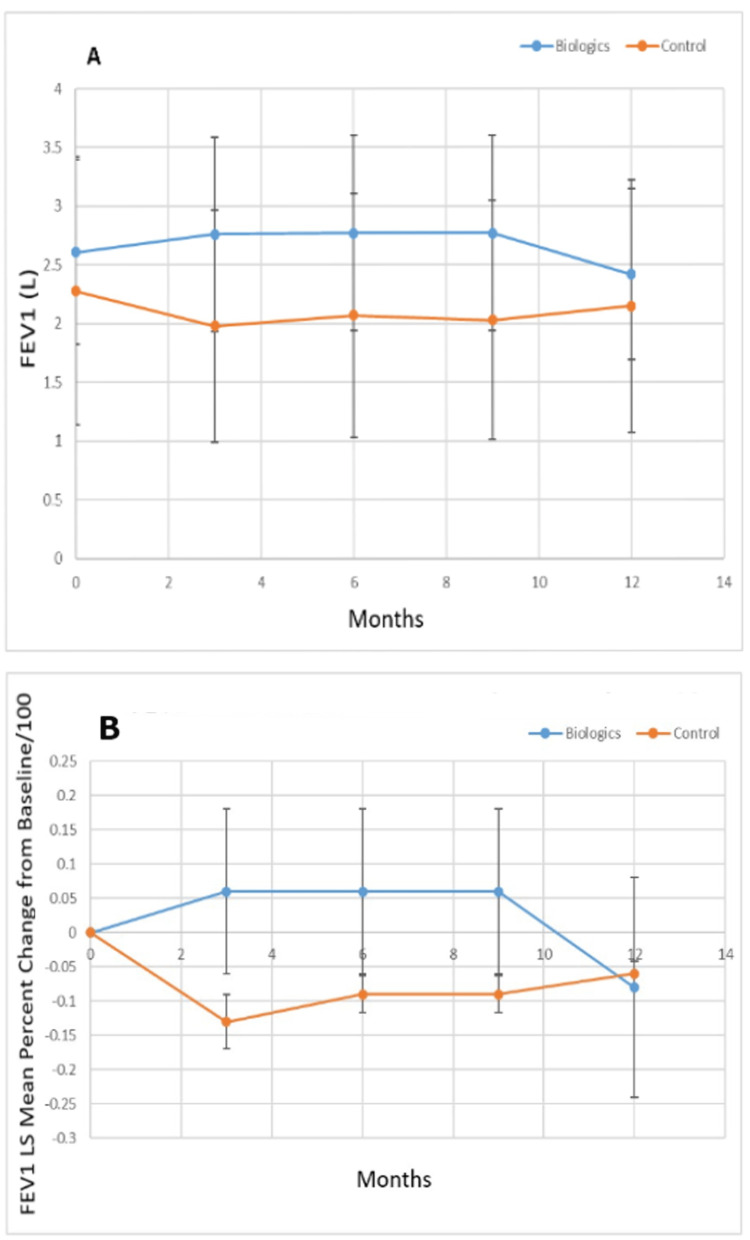
A. Least squares mean FEV1 (Liters) over the study period for biologic versus control group and B. percent change of the least squares mean of FEV1 from baseline over the study period for biologic versus control group. FEV1: Forced expiratory volume in the first second; L: Liters; LS: Least squares.

Figure 2A shows the FEV1 (Liters) for the biologic versus control groups, while Figure 2B shows the percent change from the baseline of the least squares mean value for FEV1 for the biologic and control groups. The control group had a lower baseline FEV1 (p<0.0001), but the FEV1 for the biologic treatment group was higher at three (p<0.0001), six (p<0.0001), nine (p<0.0001), and twelve (p=0.014) months. There was a statistically significant improvement (increase) in the least squares mean percent change in FEV1 for the biologic group vs. the control group at three (p<0.0001), six (p=0.0014), and nine months (p<0.001). At twelve months (p<0.0001), the least squares mean percent change from baseline for FEV1 was decreased for the biologic group.

**Figure 3:**
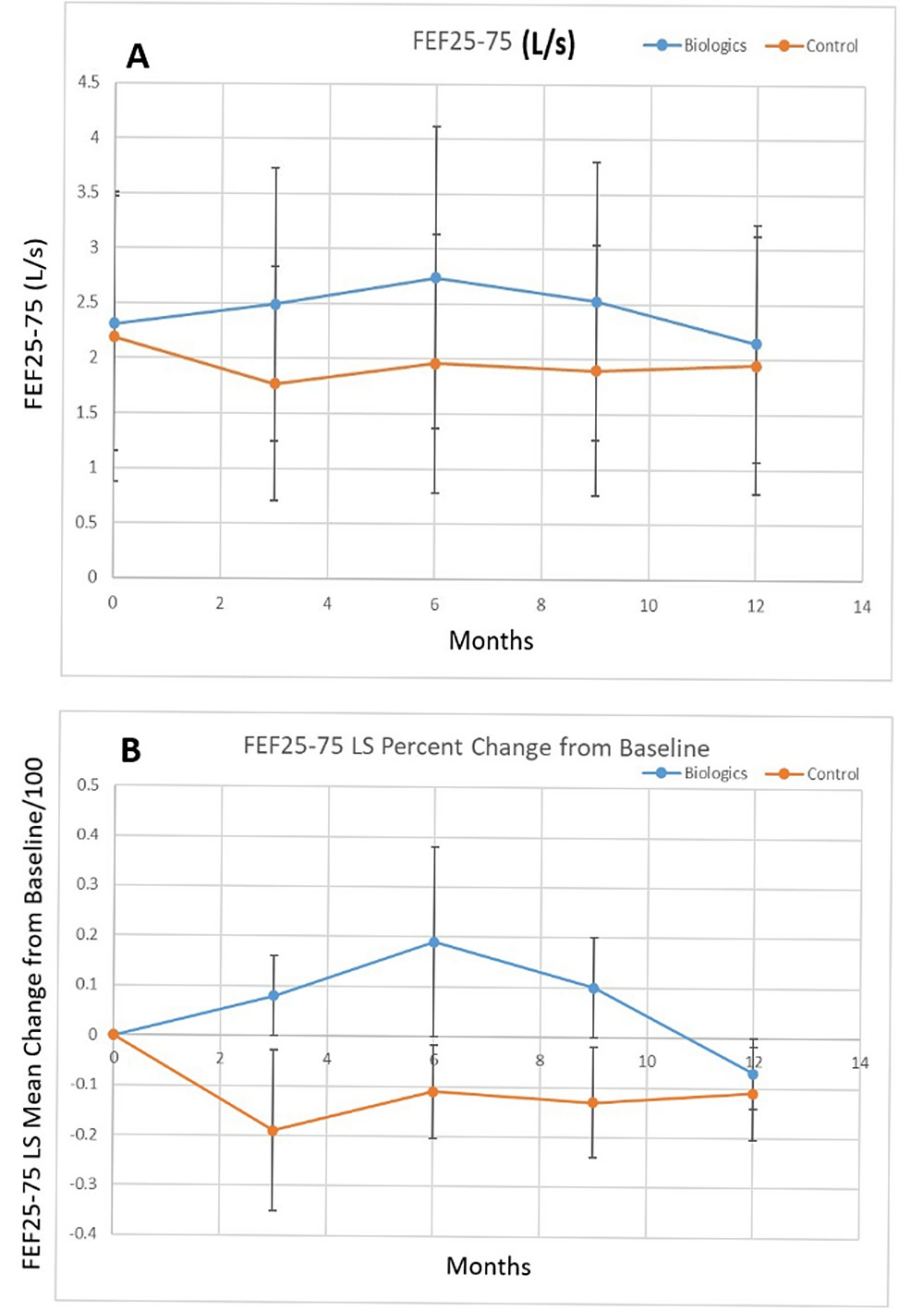
A. Least squares mean FEF25-75 (Liters/second) over the study period for biologic versus control group and B. percent change of the least squares mean of FEF25-75 from baseline over the study period for biologic versus control group. FEF25-75: Forced expiratory flow from 25% to 75% of the forced vital capacity (FVC) curve; FVC: Forced vital capacity; L/s: Liters per second (Liters/second); LS: Least squares.

Figure 3A shows the FEF25-75 (Liters/second) for the biologic versus control groups, while Figure 3B shows the percent change from the baseline of the least squares mean value for FEF25-75 for the biologic and control groups. The control group had a lower baseline FEF25-75 (p<0.0001), but the FEF25-75 for the biologic treatment group was higher at three (p<0.0001), six (p<0.0001), nine (p<0.0001), and twelve (p<0.0001) months. There was a statistically significant improvement in the least squares mean percent change in FEF25-75 for the biologic group vs. the control group at three (p<0.0001), six (p<0.001)) and nine months (p<0.001). At twelve months (p<0.0001) the least squares mean percent change from baseline was decreased for the biologic group vs. three through nine months but still greater than that of the control group.

**Figure 4:**
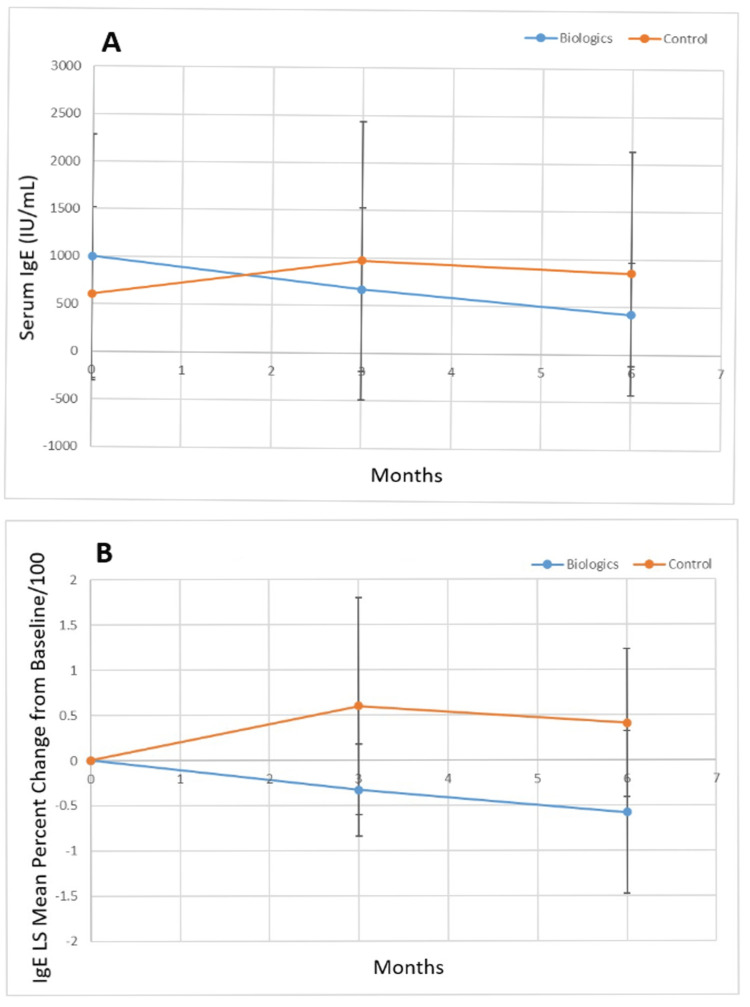
A. Least squares mean serum IgE (IU/mL) over the study period for biologic versus control group and B. percent change of the least squares mean of IgE from baseline over the study period for biologic versus control group. LS: Least squares; IgE: Immunoglobulin E; IU: International units; mL: milliliter.

Figure 4A shows the serum IgE (IU/milliliter) for the biologic versus control groups, while Figure 4B shows the percent change from the baseline of the least squares mean value for serum IgE for the biologic and control groups. The biologic group had a higher baseline serum IgE level, but this was not statistically significant (p=0.22). At three (p=0.11) and six months (p=0.0018), the serum IgE level of the biologic treated group was lower than that of the control group. Additionally, there was a statistically significant improvement (decrease) in the percent change of the least squares mean serum IgE for the biologic group vs. the control group at three and six months (p<0.0001).

**Figure 5:**
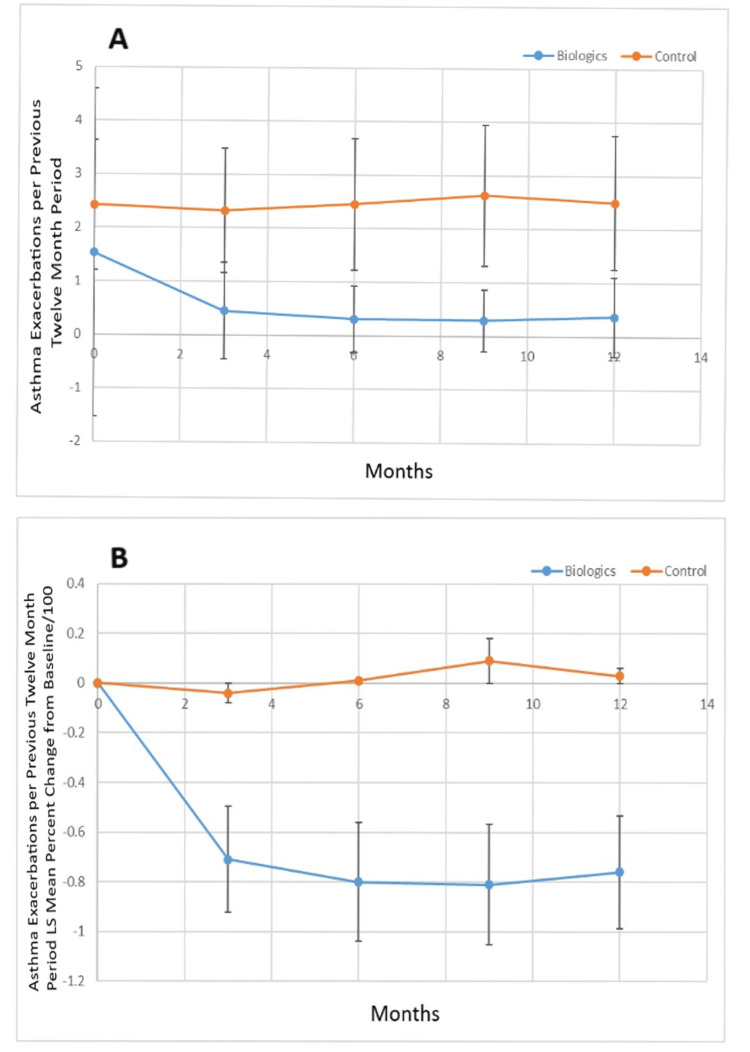
A. Least squares mean annualized pulmonary exacerbations over the study period for biologic versus control group and B. percent change of the least squares mean of annualized pulmonary exacerbations from baseline over the study period for biologic versus control group. LS: Least squares.

Figure 5A shows the annualized number of pulmonary exacerbations for the biologic versus control groups, while Figure 5B shows the percent change from baseline of the least squares mean value for annualized pulmonary exacerbations for the biologic and control groups. The biologic group had a lower baseline annualized rate of pulmonary exacerbations (p<0.0001), and the rate of annualized pulmonary exacerbations was lower for the biologic group vs. the control group at three, six, nine, and twelve months (p<0.0001). There was a statistically significant decrease in the percent change of the least squares mean annualized pulmonary exacerbations for the biologic group vs. the control group at three, six, nine, and twelve months (p<0.0001).

**Figure 6:**
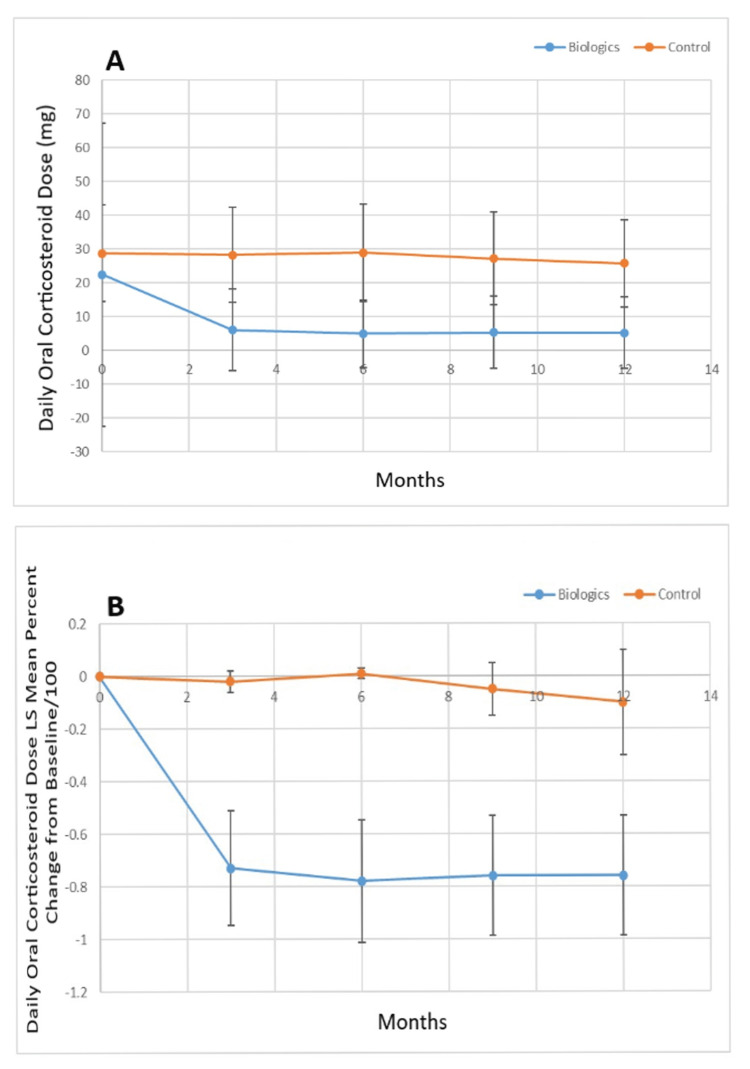
A. Least squares mean oral corticosteroid dose (mg) over the study period for biologic versus control group and B. percent change of the least squares mean of oral corticosteroid dose from baseline over the study period for biologic versus control group. LS: Least squares; mg: milligrams.

Figure 6A shows the oral daily corticosteroid dose (mg) for the biologic versus control groups, while Figure 6B shows the percent change from baseline of the least squares mean value for the oral corticosteroid dose for the biologic and control groups. The biologic group had a lower baseline mean steroid dose (p=0.03). The mean steroid dose for the biologic group was lower at three, six, nine, and twelve months vs. the control group (p<0.0001). There was a statistically significant decrease in the least squares mean oral corticosteroid dose percent change from baseline for the biologic group vs. the control group at three, six, nine, and twelve months (p<0.0001) for both analyses.

**Figure 7:**
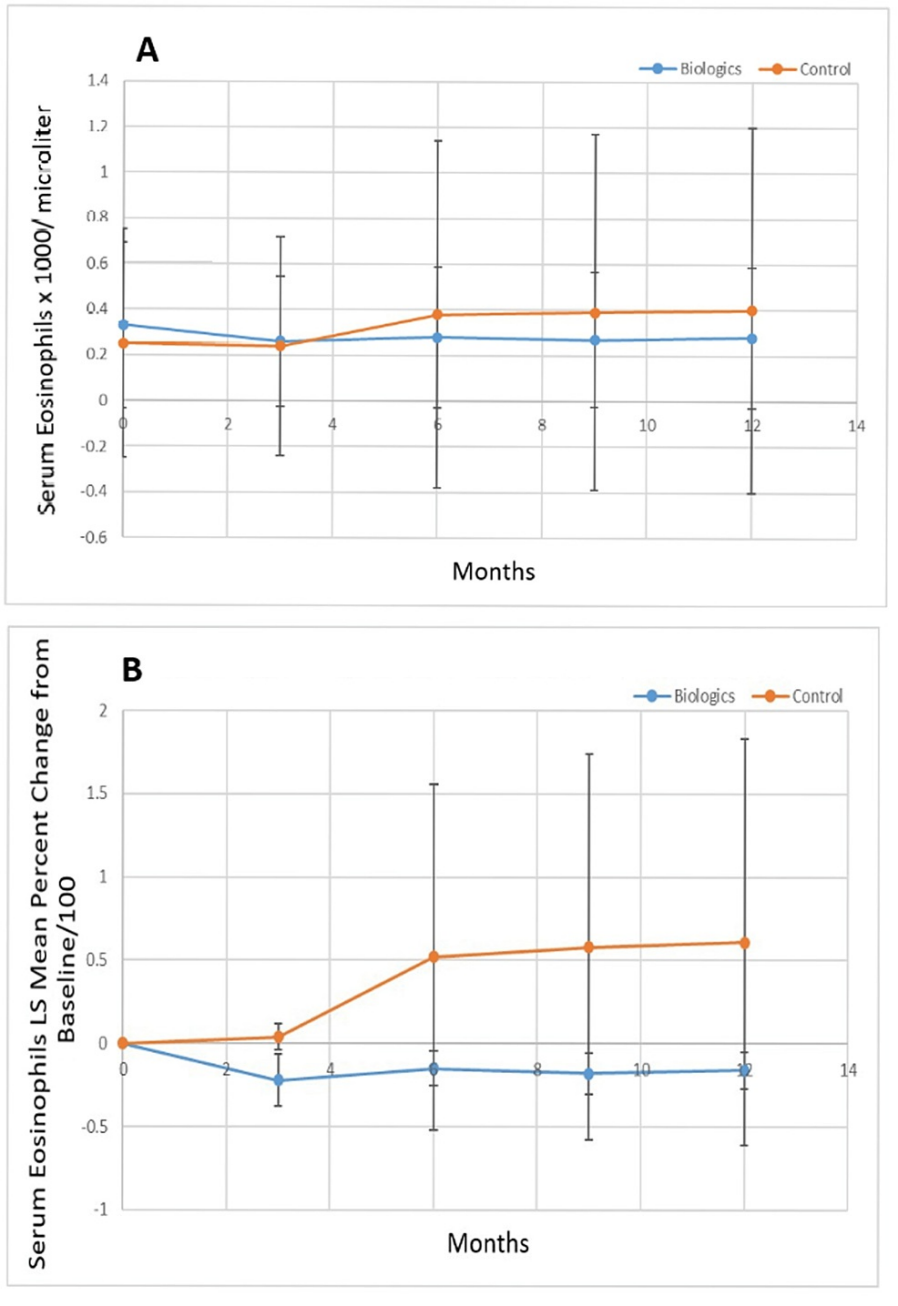
A. Least squares mean serum eosinophils (x1000/microliter) over the study period for biologic versus control group and B. percent change of the least squares mean of serum eosinophils from baseline over the study period for biologic versus control group. LS: Least squares.

Figure 7A shows the serum eosinophils (x1000/microliter) for the biologic versus control groups, while Figure 7B shows the percent change from baseline of the least squares mean value for the serum eosinophils for the biologic and control groups. The serum eosinophil level was higher at baseline for the biologic group vs. the control group (p=0.16) although this difference was not statistically significant. The serum eosinophil level was slightly higher for the biologic group vs. the control group at three months (p=0.69). At six (p=0.24), nine (p=0.094), and twelve months (p=0.10) the serum eosinophil level was lower for the biologic group, but this did not reach statistical significance. There was a statistically significant improvement (decrease) in the percent change of the least squares mean serum eosinophil level for the biologic group vs. the control group at three, six, nine, and twelve months (p<0.0001).

## Discussion

This study demonstrated significant decreases in Lund-MacKay score, pulmonary exacerbations, oral corticosteroid dose, serum IgE, and serum eosinophils for patients treated with the biologics omalizumab, benralizumab, mepolizumab, and dupilumab vs. control patients not treated with biologics. Additionally, patients treated with biologics demonstrated improved FEV1 and FEF25-75 versus controls. The control group was significantly younger than the biologic treatment group, but other demographic factors such as race, BMI, and primary diagnosis were similar between the two groups. Interestingly, the biologic treatment group had a higher baseline Lund-MacKay score, serum IgE, and eosinophil level and still showed a decrease in these factors vs. the control group.

These results are consistent with other studies that have demonstrated improved FEV1 and decreased need for oral corticosteroids in patients with asthma [9]. Similarly, omalizumab, mepolizumab, benralizumab, dupilumab, and other biologics have been shown to decrease nasal polyp scores, Lund-MacKay scores, and SNOT-22 in patients with CRSwNP, as well as decreasing the frequency of pulmonary exacerbations in patients with asthma [10]. Chapman et al demonstrated that mepolizumab treatment in patients previously treated with omalizumab decreased serum eosinophil counts in patients with severe eosinophilic asthma, similar to the finding in the present study that serum eosinophils were decreased in the biologic treatment group versus the control group [16]. In their meta-analysis of the effectiveness of omalizumab in patients with allergic asthma Bousquet et al found that omalizumab improved FEV1, reduced exacerbations, and reduced oral corticosteroid use versus controls [17]. This is similar to the results seen in the present study in which the biologic treatment group also experienced a significant improvement in prebronchodilator FEV1 and significant decreases in annualized asthma exacerbations and oral corticosteroid dose vs. controls. Mullol et al found that dupilumab improved FEV1 and decreased Lund-Mackay computed tomography (CT) scores and disease-related events/exacerbations, while also improving a number of olfactory and nasal function outcome measures in patients with aspirin-exacerbated respiratory disease and refractory chronic sinusitis with sinonasal polyposis [18]. The present study similarly noted improvements in FEV1 and reductions in the Lund-Mackay score, decreased pulmonary exacerbations, and reduced corticosteroid use in the biologic treatment group.

This study does have limitations. The age difference in the biologic and control groups and the different proportions of patients with chronic sinusitis with sinonasal polyposis in the biologic and treatment groups may affect the outcome measures reported and their comparison. The retrospective nature introduces the risk of selection bias. Additionally, as this was not a prospective trial there was significant missing data, with sufficient data on Lund-MacKay score not available for six and nine months, for serum IgE not available for months nine and twelve, and insufficient analyzable data on SNOT-22, ACQ-5, ACT, NOSE, need for FESS, and FENO. Prospective studies with randomization and standardized collection of pulmonary function tests, maxillofacial CT scans, serum IgE and complete blood counts with differential, and collection of SNOT-22, ACQ-5, ACT, and NOSE scores at regular intervals would provide useful increased data on the effect of biologic treatment in patients with asthma, allergic rhinitis, and chronic rhinosinusitis with sinonasal polyposis.

## Conclusions

This retrospective study demonstrated that patients treated with the biologics omalizumab, benralizumab, mepolizumab, and dupilumab showed decreased Lund-MacKay radiographic scores, decreased serum IgE and eosinophil levels, decreased pulmonary exacerbations, and decreased oral corticosteroid dose versus control patients. Additionally, patients treated with biologics showed improved prebronchodilator FEV1 and FEF25-75 on pulmonary function tests versus controls. These results correlate well with previous prospective trials and meta-analyses in the literature showing similar pulmonary and sinus symptoms and functional status improvements for patients treated with biologics for type 2 inflammatory diseases. For patients who are candidates for biologic treatment, these medications can provide significant benefits.
